# The GTPase-Activating Protein FgGyp1 Is Important for Vegetative Growth, Conidiation, and Virulence and Negatively Regulates DON Biosynthesis in *Fusarium graminearum*

**DOI:** 10.3389/fmicb.2021.621519

**Published:** 2021-01-21

**Authors:** Qiaojia Zheng, Zhi Yu, Yanping Yuan, Danli Sun, Yakubu Saddeeq Abubakar, Jie Zhou, Zonghua Wang, Huawei Zheng

**Affiliations:** ^1^Marine and Agricultural Biotechnology Laboratory, Institute of Oceanography, Minjiang University, Fuzhou, China; ^2^College of Life Sciences, Fujian Agriculture and Forestry University, Fuzhou, China; ^3^Department of Biochemistry, Faculty of Life Sciences, Ahmadu Bello University, Zaria, Nigeria

**Keywords:** *Fusarium graminearum*, GTPase-activating protein, FgGyp1, DON, fungal development, pathogenicity

## Abstract

Ypt1 is a small Rab GTPase in yeast, Gyp1 functions at the Golgi as a negative regulator of Ypt1. Gyp1 homologs are conserved in filamentous fungi. However, the roles of Gyp1 in phytopathogenic fungi are still unclear. Herein, we investigated the functions of FgGyp1 in the wheat pathogen *Fusarium graminearum* by live-cell imaging, genetic, and pathological analyses. Targeted gene replacement method was used to delete *FgGYP1* in *F. graminearum*. Phenotypic analyses showed that FgGyp1 is critically important not only for the vegetative growth of *F. graminearum* but also its conidiation. The mutant’s vegetative growth was significantly reduced by 70% compared to the wild type PH-1. The virulence of *FgGYP1* deletion mutant was significantly decreased when compared with the wild type PH-1. We further found that FgGyp1 negatively regulates DON production of the fungus. Live-cell imaging clearly demonstrated that FgGyp1 mainly localizes to the Golgi apparatus. Moreover, the TBC domain, C-terminal, and N-terminal regions of FgGyp1 are found to be indispensable for its biological functions and normal localization. The Arg357 residue of FgGyp1 is essential for its functions but dispensable for the normal localization of the protein, while the Arg284 residue is not required for both the functions and normal localization of the protein. Furthermore, we showed that FgGyp1 essentially hydrolyzes the GTP-bound FgRab1 (activated form) to its corresponding GDP-bound (inactive) form *in vitro*, suggesting that FgGyp1 is a GTPase-activating protein (GAP) for FgRab1. Finally, FgGyp1 was found to be important for FgSnc1-mediated fusion of secretory vesicles from the Golgi with the plasma membrane in *F. graminearum*. Put together, these data demonstrate that FgGyp1 functions as a GAP for FgRab1 and is important for vegetative growth, conidiation and virulence, and negatively regulates DON biosynthesis in *F. graminearum*.

## Introduction

*Fusarium graminearum* causes the Fusarium head blight (FHB) of wheat, barley and other cereals, globally (McMullen et al., 1997; Bushnell et al., 2003). FHB epidemics have been prevalent in China since 2010 and caused enormous yield losses so far (Chen et al., 2019). Furthermore, *F. graminearum* infestation has serious negative impacts on human and animal health as the pathogen renders the crops contaminated with mycotoxins, such as zearalenone (ZEA) and trichothecenes, which when consumed cause serious food poisoning (Bushnell et al., 2003; Chen et al., 2019). In recent years, a number of studies demonstrated that vesicles trafficking is indispensable for the fungal normal development, pathogenicity, and DON production (Zheng et al., 2015, 2018b,c; Zhang et al., 2016; Li et al., 2017, 2018, 2019; Xie et al., 2019; Adnan et al., 2020).

Small Rab GTPases regulate vesicle trafficking processes in eukaryotic cells, including endocytosis and exocytosis (Li and Marlin, 2015). GTPase-activating proteins (GAPs) are negative regulators of Rab GTPases, they do so by inactivating Rab GTPases *via* promoting their GTPase activity (Mizuno-Yamasaki et al., 2012). As regulators of Rab-dependent pathways, RabGAPs regulate many diseases including bacterial and viral infections, development of cancer, and onset of obesity in mammalian cells (Mizuno-Yamasaki et al., 2012). So far, most identified RabGAPs contain Tre2-Bub2-Cdc16 (TBC) domains (Fukuda, 2011). Gyp1 was previously shown to regulate the trafficking of vesicles from the ER (endoplasmic reticulum) to the plasma membrane (Huang et al., 2014). Gyp1 interacts *in vitro* with Ypt1, Ypt7, Ypt51, and Sec4 (Du et al., 1998; Albert and Gallwitz, 1999). As a GAP of the small GTPase Ypt1, Gyp1 negatively regulates Ypt1 on the Golgi membrane (Du and Novick, 2001). The protein is also a GAP for Sec4p, a protein involved in the secretion pathway (Du et al., 1998). It has an arginine residue in its motif B sequence and such residue is essential for its catalytic function (Albert and Gallwitz, 1999). Furthermore, the crystal structure of Gyp1 GAP domain reveals important insights into the mechanism of its interaction with Ypt/Rab proteins, showing that in the GAP reaction Gyp1 uses an arginine finger similar the interactions of Cdc42-GAP and Ras-GAP (Rak et al., 2000).

In budding yeast, Gyp1 is dispensable for cell viability when grown on nutrient-rich media at different temperatures (Du et al., 1998) while Δ*gyp1* mutant shows defect in growth on a medium at 37°C (Du and Novick, 2001). In addition, the *gyp1* deletion mutant showed minimal medium-specific growth defect (Winzeler et al., 1999). A study in *Candida albicans* demonstrates that Gyp1 is required for hyphal growth and Golgi polarization, and it localizes to Golgi apparatus (Huang et al., 2014). However, the functions of Gyp1 in other filamentous fungi particularly in *F. graminearum* remain obscured.

In our previous study, we systematically characterized all the 11 putative Rab proteins in *F. graminearum* and found that they are essentially required for growth and pathogenicity of *F. graminearum* (Zheng et al., 2015). FgMon1, FgSec2A, and FgVps9 proteins are GEFs (Guanine nucleotide exchange factors) for FgRab7, FgRab8, and FgRab5, respectively (Li et al., 2015; Zheng et al., 2018a; Yang et al., 2020). They are all required for pathogenicity and development of *F. graminearum*. Recently, we demonstrated that FgMsb3 protein is a GAP for FgRab8 and regulates hyphal tip expansion, polarized trafficking and pathogenicity in *F. graminearum* (Zheng et al., 2020).

FgRab1 is critically important for *F. graminearum* normal development (Zheng et al., 2015), but the upstream regulators of FgRab1 are still unknown. The fact that Gyp1 serves as a GAP of Ypt1/Rab1 in yeast prompted us to dissect the relationship between FgGyp1 and FgRab1, as well as the function(s) of FgGyp1 in *F. graminearum*. Our *in vitro* analyses indicate that FgGyp1 is a GAP of FgRab1 and is important for growth, conidiation, virulence, and DON biosynthesis of *F. graminearum*.

## Materials and Methods

### Strains Used and Culture Conditions

The Supplementary Table 1 presents all the strains used in this study. The culture media used in the study include complete medium (CM) consisting (all in w/v) 0.6% casein acid hydrolysate, 0.6% yeast extract, 1% sucrose, 2% agar (for solid media) or starch yeast medium (SYM) consisting (all in w/v) 1% starch, 0.2% yeast extract, 0.3% sucrose, and 2% agar (Zheng et al., 2015). The incubations were done at 28 °C for 3 days. Previous protocols (Cappellini and Peterson, 1965; Zheng et al., 2015; Fan et al., 2020) were used to assay for conidiation and sexual reproduction.

### *FgGYP1* Gene Disruption and Complementation

Previously reported protocols (Hou et al., 2002) were used for the preparation of *F. graminearum* protoplasts and subsequent transformations. The split-marker approach was used to generate gene replacement constructs for the *FgGYP1* (FGSG_17336) deletion mutants. The Supplementary Table 2 presents all the primers used for this purpose. Two gene deletion mutants (Δ*Fggyp1*) were successfully generated and further confirmed by Southern blotting. To reintroduce this gene into the mutant (complementation), a vector harboring FgGyp1-GFP fusion protein was generated by amplifying the coding sequence and native promoter of FgGyp1 using FgGyp1CF and FgGyp1CR primer pair (Supplementary Table 2). A Cloning Kit (Vazyme Biotech Co., Ltd., China) was then used to insert the amplicon into a pKNTG2 vector and the product was sequenced for verification. The vector (pFgGyp1-GFP) was then transformed into the protoplast of Δ*Fggyp1* mutant and a complemented strain was successfully generated which has similar phenotypes to the PH-1.

### Constructions of pFgBet3-mCherry, pFgGyp1^Δ^*^*N*^-*GFP, pFgGyp1^Δ^*^*TBC*^*-GFP, pFgGyp1^Δ^*^*C*^*-GFP, pFgGyp1^*R357K*^ (R357K), and pFgGyp1^*R284K*^ (R284K) Point Mutation Vectors

The pFgBet3-mCherry vector was constructed by amplification of the 2,573-bp FgBet3 sequence (including its native promoter and coding sequence) using FgBet3CF and FgBet3CR primer pair (Supplementary Table 2). The previously stated cloning kit was used to clone the PCR product into a pKNT-mCherry plasmid and the insertion was confirmed by sequencing. pFgGyp1^Δ^*^*N*^-*GFP, pFgGyp1^Δ^*^*TBC*^*-GFP, pFgGyp1^Δ^*^*C*^*-GFP and pR357K, pR284K constructs were generated by PCR amplification of the sequences using their respective primer pairs (Supplementary Table 2) and pKNTG2 vector was used for the cloning and verified by sequencing.

### Pathogenicity and Deoxynivalenol Production Assays

Flowering wheat heads were used to test for the pathogenicity of the various strains as previously reported (Zheng et al., 2015). Wheat coleoptiles were also used for this assy. Briefly, 2 μl of 100 × 10^4^ cells/ml conidial suspension was inoculated in each coleoptile and the observed symptoms were recorded after 7 days of inoculation. To investigate their DON production abilities, the various strains were cultured in TBI (trichothecene biosynthesis induction) liquid media and incubated for 7 days in the dark at 28°C without shaking, after which the mycelia were separated from the liquid (Gardiner et al., 2009). An ELISA (enzyme linked immunosorbent assay)-based DON assay kit (Beacon Analytical Systems, Saco, ME, United States) was used to check for DON levels in the liquids while the mycelia were quantified after drying. The experiment was repeated three times.

### GTPase Activity Assay

The sequences of FgRab1 cDNA, FgGyp1 cDNA, TBC domain and R357K point mutant of FgGyp1 were cloned into the MBP (Maltose-binding protein) vector pMAL-c2X, using their respective primers listed in Supplementary Table 2, and expressed. The protein products were isolated and purified for GAP activity assay using a GTPase assay kit (Sigma-Aldrich, Catalog Number MAK113) according to the manufacturer’s protocols. GTP hydrolysis was assayed based on previous reports (Xiong et al., 2012; Zheng et al., 2020). Briefly, the recombinant proteins MBP–FgGyp1, MBP–FgGyp1^*TBC*^ (TBC), MBP–FgGyp1^*R357K*^ (R357), and MBP–FgRab1 were expressed in BL21 *Escherichia coli* strain and isolated by Amylose resin (Sangon Biotech, NO.C500096) as per the instructions of the manufacturer. A colorimetry-based kit was used (Sigma-Aldrich, Catalog Number MAK113) to assay for the GTPase activity of Gyp1, and FgRab1 was incubated with FgGyp1, FgGyp1^*TBC*^ (TBC), FgGyp1^*R357K*^ (R357K), and MBP control, respectively, following the instructions of the manufacturer. The experiment was repeated three times.

### Accession Numbers

*Aspergillus fumigatus* (AfGyp1-XP_749737.1), *Candida albicans* (CaGyp1-XP_717292.1), *Fusarium graminearum* (FgGyp1-PCD18761.1), *Fusarium oxysporum* (FoGyp1-EMT63394.1), *Fusarium verticillioides* (FvGyp1-EWG40605.1), *Magnaporthe oryzae* (MoGyp1-ELQ43659.1), *Neurospora crassa* (NcGyp1-CAC18313.2), *Saccharomyces cerevisiae* (ScGyp1-NP_014713.1), *Schizosaccharomyces pombe* (SpGyp1-NP_595314.1), and *Ustilago maydis* (UmGyp1-XP_011391412.1).

### Live Cell Imaging of *Fusarium graminearum*

The live cell imaging of *F. graminearum* mycelia and conidia was performed using an Olympus BX51 (Olympus, Japan) microscope and a laser confocal microscope (Nikon, Japan). The following settings were used. GFP excitation: 488 nm light (Em.525/40 nm); mCherry excitation: 561 nm light (Em. 607/36 nm).

## Results

### *FgGYP1* Gene Deletion and Confirmation

To identify the *FgGYP1* gene in *F. graminearum* genome, we used the budding yeast Gyp1 amino acid sequences as a reference to carry out a BLAST search against the available fungal genomes. We were able to identify a homolog of Gyp1 at the FGSG_17336 locus of *F. graminearum* genome. FGSG_17336 encodes a protein of 601 amino acid residues and it is 40.53% similar to the yeast Gyp1, and it covers 61.00% of the total length of FgGyp1 and ScGyp1. The phylogenetic relationship of Gyp1 homologs suggest that Gyp1 is conserved in plant pathogenic fungi, especially in *Fusarium oxysporum*, *Fusarium verticillioides*, *Neurospora crassa*, and *Magnaporthe oryzae* (Supplementary Figure 1). Furthermore, targeted gene replacement strategy was used to delete *FgGYP1* gene in PH-1 (wild type) where many positive mutants were identified by PCR screening (Supplementary Figure 2A). Two of these mutants (Δ*Fggyp1-2* and Δ*Fggyp1-5*) were subjected to Southern blotting for confirmation (Supplementary Figure 2B).

### FgGyp1 Plays an Important Role in Vegetative Growth and Conidiation

**FIGURE 1:**
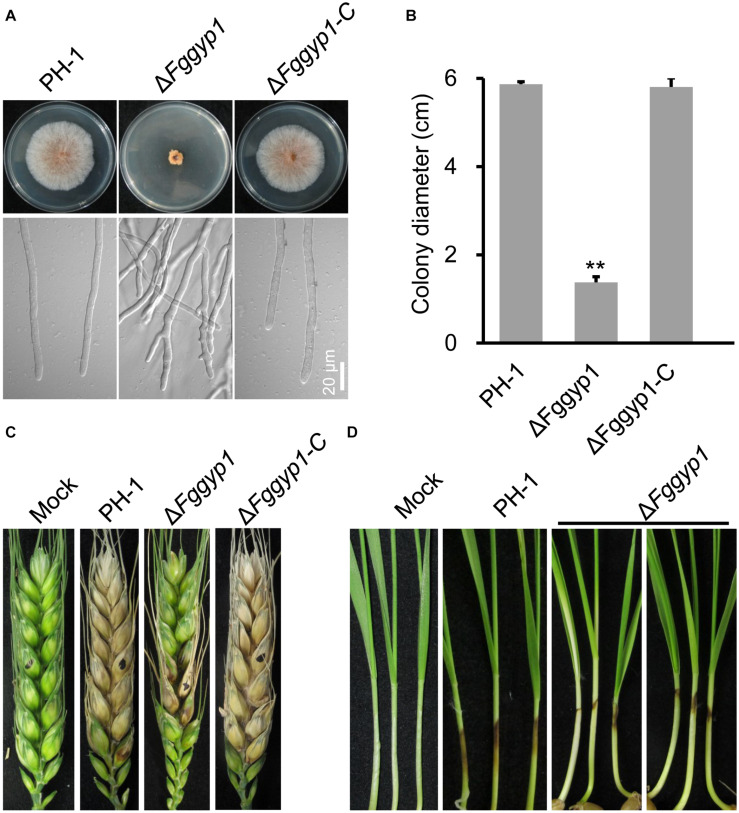
FgGyp1 is required for vegetative growth and virulence of *Fusarium graminearum*. **(A)** Colonies morphology of the wild type (PH-1), Δ*Fggyp1* mutant, and Δ*Fggyp1-C* (the complemented strain) after growth on CM agar for 3 days. **(B)** Statistical analysis of the colony diameters of the PH-1, Δ*Fggyp1*, and Δ*Fggyp1-C* strains on CM medium after 3 days. Error bars represent the standard deviation from three replicates and Two-tailed Student’s *t*-test was used for paired comparison of the colony diameters between Δ*Fggyp1* mutant and the wild type PH-1 strain (**P < 0.01). **(C)** Pathogenicity test of PH-1, Δ*Fggyp1*, and Δ*Fggyp1-C* strains inoculated on wheat heads for 14 days. The virulence of Δ*Fggyp1* mutant is significantly reduced. **(D)** Pathogenicity of PH-1, Δ*Fggyp1*, and Δ*Fggyp1-C* strains on wheat coleoptiles after 7 days of inoculation. The virulence of Δ*Fggyp1* mutant is significantly decreased.

To determine whether FgGyp1 is required for the fungal vegetative growth, we cultured the Δ*Fggyp1* mutant on CM (complete media) and monitored its growth rate after 3 days. Compared to the PH-1 and Δ*Fggyp1-C* (complemented strain) strains, the Δ*Fggyp1* growth rate was critically reduced (Figures 1A,B and Table 1). Close microscopic examinations indicated numerous branching in the Δ*Fggyp1* mutant hyphae which was not observed in PH-1 and Δ*Fggyp1-C* strains (Figure 1A). These results suggest that FgGyp1 is important for *F. graminearum* vegetative growth and hyphal polarity.

**TABLE 1:** Phenotypic characterization of Δ*Fggyp1* mutant.

**Strain**	**Colony diameter**	**Conidiation**	**Disease**	**DON**
	**(cm)^1^**	**(× 10^4^/ml)^2^**	**index^3^**	**(mg/g)^4^**
PH-1	5.87 ± 0.06	192.63 ± 3.94	13.22 ± 2.76	29.45 ± 7.61
Δ*Fggyp1*	1.38 ± 0.13**	50.13 ± 5.32**	5.88 ± 1.85**	68.34 ± 4.61*
Δ*Fggyp1-C*	5.81 ± 0.20	184.88 ± 27.28	13.33 ± 2.68	32.34 ± 8.45

^1^Colony diameter was measured after incubating on CM agar plates for 3 days. ^2^Conidiation was measured by counting the number of conidia in CMC media for 3 days. ^3^Disease index was rated by the number of symptomatic spikelet after inoculation in field for 2 weeks. ^4^DON was determined in liquid TBI media at 28°C for 7 days. Mean and standard error were calculated from three independent measurements. Two-tailed Student’s *t*-test was used for paired comparison between Δ*Fggyp1* mutant vs. wild type PH-1 (**P < 0.01; *P < 0.05).

The major *F. graminearum* inoculums that infect flowering wheat heads are the conidia (Francl et al., 1999). As such, we inoculated the wild type PH-1, Δ*Fggyp1*, and Δ*Fggyp1-C* strains on carboxymethylcellulose (CMC) media at 28°C for 3 days for conidia production. As shown in Table 1, the conidiation of Δ*Fggyp1* mutant decreased significantly when compared to those produced by PH-1 and Δ*Fggyp1-C* strains, suggesting that FgGyp1 plays an important role in the fungal conidiogenesis. In addition to conidia, the ascospores of *F. graminearum* are also an important inoculum in its infection cycle. However, sexual reproduction and ascospore formation are not significantly affected by *FgGYP1* deletion (Supplementary Figures 3A,B).

### FgGyp1 Is Required for Virulence and Negatively Regulates the Biosynthesis of DON

To investigate the effect of *FgGYP1* deletion on the pathogenicity of the fungus, the mutant and the controls were inoculated on flowering wheat heads under moist condition for 14 days. As shown in Figure 1C, deletion of *FgGYP1* gene caused significantly decreased infection capability to wheat heads. The average disease index (diseased spikelets per head) for Δ*Fggyp1* mutant is less than six while the disease index of PH-1 and Δ*Fggyp1-C* strains are more than 13 (Table 1). Furthermore, infection assays on wheat coleoptiles yielded similar results (Figure 1D). Taken together, our data here demonstrate that FgGyp1 is important for *F. graminearum* virulence.

**FIGURE 2:**
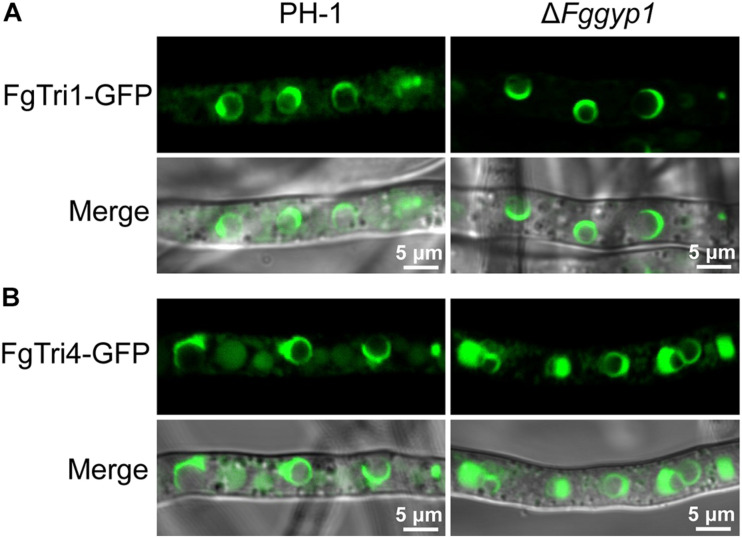
FgGyp1 is dispensable for FgTri1/FgTri4-mediated toxisome formation. **(A)** Localization of FgTri1-GFP protein in the PH-1 and Δ*Fggyp1* mutant. Deletion of *FgGYP1* did not affect the formation of FgTri1-GFP-labeled toxisome. **(B)** Localization of FgTri4-GFP protein in PH-1 and Δ*Fggyp1* mutant. Deletion of *FgGYP1* did not affect the formation of FgTri4-GFP-labeled toxisome.

In *F. graminearum*, deoxynivalenol (DON) has been widely studied as an important mycotoxin and virulence factor (Proctor et al., 1995). To investigate whether FgGyp1 is required for DON production, we checked and compared the levels of DON in the PH-1, Δ*Fggyp1*, and Δ*Fggyp1-C* strains cultured in liquid TBI media for 7 days at 28°C in the dark. We found that Δ*Fggyp1* mutant showed strikingly higher level of DON production than the wild type PH-1 (Table 1, the standard curves for quantification of the DON were showed in Supplementary Figure 4), suggesting that FgGyp1 negatively regulates DON biosynthesis in *F. graminearum*. However, we found that the trichothecene biosynthesis proteins FgTri1-GFP and FgTri4-GFP retain their normal localizations at the toxisomes of both strains, meaning that FgGyp1 is dispensable for their localizations (Figure 2).

### FgGyp1 Localizes to the *Trans*-Golgi Network in *Fusarium graminearum*

**FIGURE 3:**
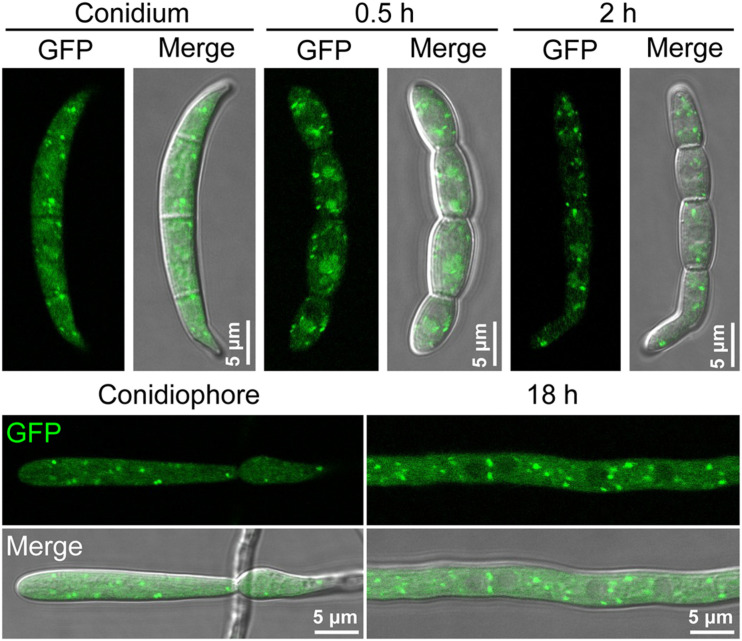
The localization of FgGyp1-GFP fusion protein in non-germinating and germinating conidia (0.5 and 2 h), mycelia (18 h), and conidiophore of *Fusarium graminearum*.

**FIGURE 4:**
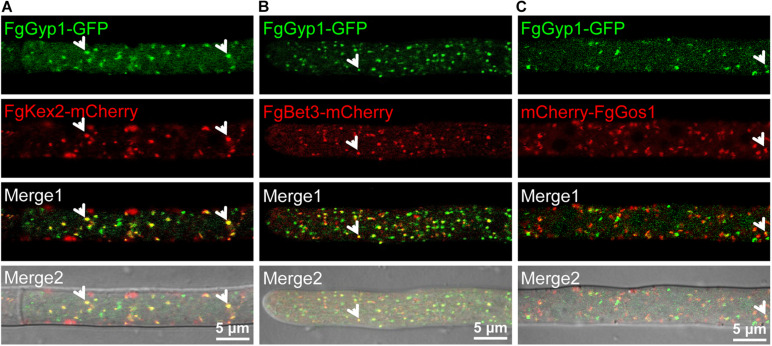
The co-localization of FgGyp1 with *trans*-Golgi network (TGN, FgKex2-mCherry), *cis*-Golgi marker (FgBet3-mCherry), and medial Golgi marker (mCherry-FgGos1) in *Fusarium graminearum*. **(A)** FgGyp1 partially co-localized with FgKex2-positive TGN (61.72 ± 4.67% co-localization). Arrows show co-localization. **(B)** FgGyp1 partially co-localized with FgBet3-positive *cis*-Golgi (28.79 ± 12.03% co-localization). Arrows show co-localization. **(C)** FgGyp1 partially co-localized with FgGos1-positive medial Golgi (14.71 ± 6.48% co-localization). Arrows show co-localization.

To establish the sub-cellular localization of FgGyp1, we generated and transformed a FgGyp1-GFP-expressing vector into the Δ*Fggyp1* mutant protoplasts. The positive transformants were then subjected to live cell confocal microscopy. As shown in Figure 3, FgGyp1-GFP fluorescence were clearly visible as puncted structures distributed through the conidia and conidiophores of the fungus at all developmental stages (0.5, 2, and 18 h). In yeast, Gyp1 localizes to the Golgi apparatus (Du and Novick, 2001). To check whether the observed puncta are localized to the Golgi apparatus, several Golgi markers were generated, including the *trans*-Golgi network (TGN) marker, FgKex2-mCherry, *cis*-Golgi marker, FgBet3-mCherry, and the medial Golgi marker, mCherry-FgGos1 (Thomas et al., 2018). These constructs were co-transformed with FgGyp1-GFP, respectively, into the Δ*Fggyp1* mutant and their intracellular localization examined by confocal microscopy. As shown in Figure 4, we found that FgGyp1-GFP partially colocalizes with FgKex2-positive TGN (61.72 ± 4.67% colocalization), FgBet3-positive *cis*-Golgi (28.79 ± 12.03% colocalization) and FgGos1-positive medial Golgi (14.71 ± 6.48% colocalization) in CM medium. We therefore conclude here that FgGyp1 mainly localizes to the TGN.

### FgGyp1 Functions as a GAP for FgRab1

**FIGURE 5:**
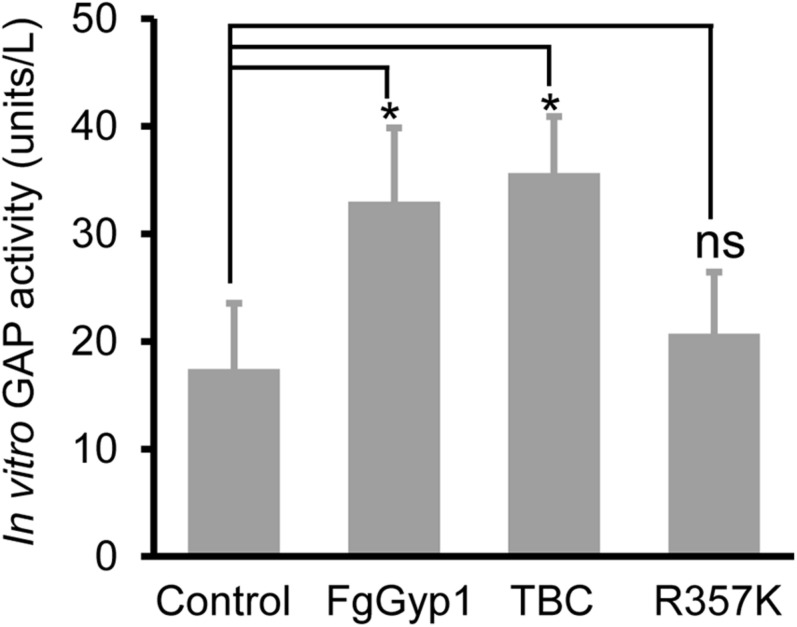
FgGyp1 functions *in vitro* as a FgRab1 GAP in *Fusarium graminearum*. *In vitro* GAP activity assay of FgGyp1. Control: MBP protein. One unit represents the amount of FgGyp1 catalyzing the release of 1 μM free phosphate/min under the experimental conditions. 1.6 μg MBP, 1.6 μg MBP-FgGyp1, 1.6 μg MBP-TBC, 1.6 μg MBP-R357K, and 0.4 μg MBP-FgRab1 proteins were used in the GAP activity assay, respectively. Two-tailed Student’s *t*-test was used for paired comparison of the GAP activity between MBP control and the full lengths of FgGyp1, TBC domain and R357K, respectively (*P < 0.05). ns, ‘no significant difference’.

Ypt1/Rab1 is a substrate for Gyp1 in yeast (Albert and Gallwitz, 1999). This prompted us to check if FgGyp1 is a GTPase-activating protein (GAP) for Rabs in *F. graminearum*. To achieve this, we investigated its Rab GAP activity *in vitro* by cloning and expressing its full-length and FgRab1 in BL21 cells, respectively. We then analyzed FgGyp1 GTP-hydrolyzing potential by quantifying the amount of phosphate moiety released by activated (GTP-bound) FgRab1. As shown in Figure 5, we found that FgGyp1 has higher efficiency in hydrolyzing GTP-bound FgRab1 than the control. Furthermore, we equally found that the TBC domain of FgGyp1 more efficiently hydrolyzes GTP-bound FgRab1 than the control. The arginine 343 residue (Arg343) of the TBC domain of Gyp1 in yeast was shown to be essential for the Gyp1 GAP activity (Du and Novick, 2001). For this reason, we identified the conserved Arginine residue in FgGyp1 (Arg357) and analyzed its role in FgGyp1 GAP activity. We observed that the FgGyp1 GAP activity was significantly decreased when Arg357 residue of FgGyp1 was mutated to lysine (R357K), as shown in Figure 5. Considering these data, we conclude that FgGyp1 catalytically converts an active (GTP-bound) FgRab1 to its inactive (GDP-bound) form through its TBC domain, and Arg357 residue is an important GTP-hydrolyzing residue of FgGyp1.

### Functional Characterizations of TBC Domain, Arg357/284 Residues and the N/C-Terminal Regions of FgGyp1

**FIGURE 6:**
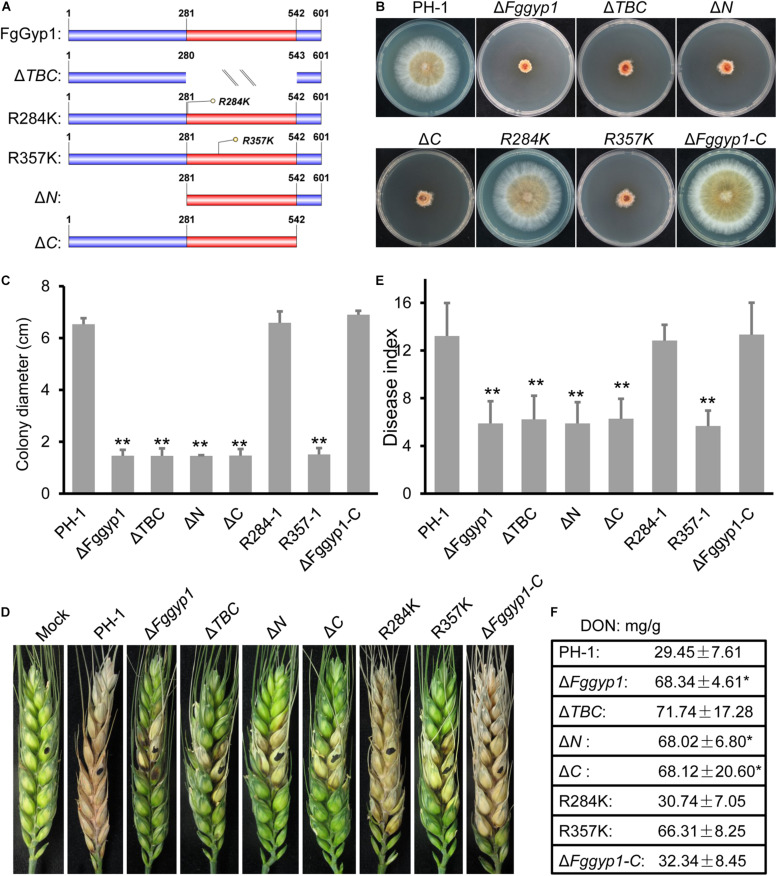
Functional characterization of TBC domain, Arg357/284 residues and the N/C-terminal regions of FgGyp1. **(A)** Schematic diagram of FgGyp1-full length, TBC domain mutant, Arg357/284 residues mutants, N-terminal, and C-terminal mutants. **(B,C)** Colony morphologies and diameters of PH-1, Δ*Fggyp1*, Δ*TBC*,Δ*N*, Δ*C*, *R284K*, *R357K*, and Δ*Fggyp1-C* strains on CM after 3 days. **(D,E)** Pathogenicity and disease indexes of the indicated strains on wheat heads. **(F)** DON production of the indicated strains in liquid TBI media. Two-tailed Student’s *t*-test was used for paired comparison of the diameter, disease index, and DON production between wild type PH-1 and the indicated strains, respectively (*P < 0.05; **P < 0.01).

Since the TBC domain (with its Arg357 residue) is required for FgGyp1 GAP activity, we set to find out to the roles of this domain as well as its Arg357 residue in the biological functions of FgGyp1 in *F. graminearum*. To unveil this, we generated the following forms of FgGyp1-GFP fusion constructs: *Fggyp1*^Δ^*^*TBC*^-GFP* (lacking the TBC domain), *Fggyp1^*R357K*^-GFP* (mutation of the arginine 357 residue with lysine), *Fggyp1^*R284K*^-GFP* (having arginine 284 replaced with lysine, control), *Fggyp1*^Δ^*^*N*^-GFP* (lacking the N-terminal aa 1–280), and *Fggyp1*^Δ^*^*C*^-GFP* (lacking the C-terminal aa 543–601) (Figure 6A). We transformed these constructs into separate protoplasts of Δ*Fggyp1* mutant and analyzed the phenotypes of each of these mutants and further examined their respective intracellular localizations by confocal microscopy. Figures 6B–F shows that Δ*TBC*, Δ*N*, Δ*C*, and *R357K* mutants exhibit phenotypes similar to the defects observed in the Δ*Fggyp1* mutant, including impaired vegetative growth, conidiation, virulence, and DON production. In contrast, the phenotypes of *R284K* mutant remain similar to those of the controls (Figures 6B–F), suggesting that Arg284 is not required for the function of FgGyp1.

**FIGURE 7:**
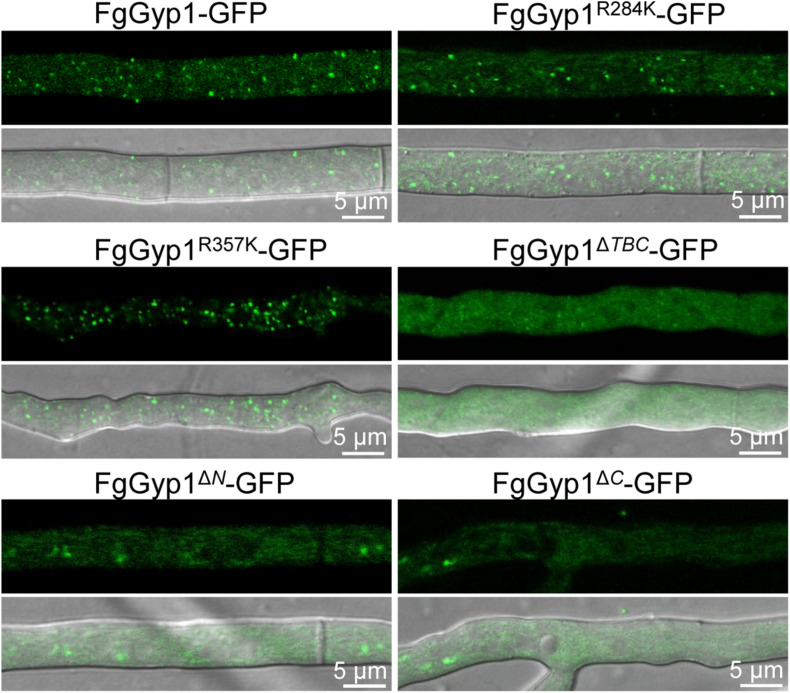
The normal localization of FgGyp1 requires its TBC domain, C-, and N-terminal regions. Mutations of Arg284 and Arg357 residues did not significantly affect the localization of FgGyp1. However, deletion of the TBC domain, N-terminal, and C-terminal regions of the protein caused the GFP signals to diffuse throughout the cytoplasm.

Moreover, the aforementioned mutants were further analyzed for their roles in the subcellular localization of FgGyp1 protein. Figure 7 depicts that Arg284 and Arg357 residues are expendable with respect to FgGyp1 localization since their deletions do not significantly affect the localization of the protein. However, deletions of TBC domain, N-terminal, and C-terminal regions of the FgGyp1 displayed diffused GFP signals in the cytoplasm instead of the normal puncted appearance, suggesting that these domains play an important role to the subcellular localization of FgGyp1. Taken together, we conclude that the TBC domain, N-terminal, and C-terminal regions are all required for FgGyp1 functions and normal localization in *F. graminearum*. Arg357 residue is equally important for the functions of FgGyp1 but not for its correct localization.

### FgGyp1 Is Important for Plasma Membrane Localization of the v-SNARE FgSnc1

**FIGURE 8:**
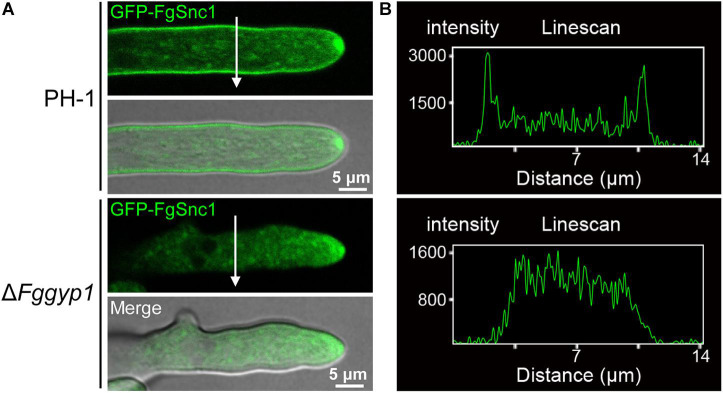
FgGyp1 is important for plasma membrane localization of the SNARE protein FgSnc1. **(A)** GFP-FgSnc1 localization in the various strains. GFP-FgSnc1 localizes to the plasma membrane of the wild type, while the membrane localization is abolished in the Δ*Fggyp1* mutant. **(B)** Line scans for GFP-FgSnc1 localization in the wild type and Δ*Fggyp1* mutant.

Membranes fusion and scission are known to be regulated by SNARE (soluble N-ethylmaleimide-sensitive factor attachment receptor) proteins during vesicle fission and delivery, respectively (Hong, 2005). In yeast, Snc1 (a SNARE protein) mediates the fusion of vesicles from the Golgi with the plasma membrane (Lewis et al., 2000). We previously demonstrated in *F. graminearum* that FgSnc1 mediates polarized secretion and fusion of vesicles (Zheng et al., 2018c). To unveil the role of FgGyp1 in vesicles fusion with the plasma membrane, we transformed GFP-FgSnc1 construct into the PH-1 and Δ*Fggyp1* protoplasts and subsequently observed their cellular localizations. Figure 8 clearly shows that in PH-1, GFP-FgSnc1 is localized on the cell membrane and accumulates at the spitzenkörper (SPK) of the growing hyphal tip (Figures 8A,B and Supplementary Video 1). In the Δ*Fggyp1* mutant, the fluorescence of GFP-FgSnc1 was similarly detected at the SPK of the growing hyphae but its plasma membrane localization was almost completely abolished (Figures 8A,B and Supplementary Video 2). These results suggest that FgGyp1 is important for FgSnc1-mediated fusion of vesicles with the plasma membrane in *F. graminearum*.

## Discussion

Rab GTPases serve as primary regulators of intracellular membrane trafficking processes (Stenmark, 2009). GTPase-activating proteins (GAPs) inactivate Rabs as when needed by hydrolyzing their GTP to GDPs (Mizuno-Yamasaki et al., 2012). Most identified RabGAPs contain Tre2-Bub2-Cdc16 (TBC) domains in mammals (Fukuda, 2011). In this study, we investigated the role of a TBC domain-containing protein FgGyp1 in the wheat pathogen *F. graminearum* and found that FgGyp1 acts as a GAP for FgRab1 *in vitro* and is importantly required for conidiation, virulence, vegetative growth and DON production in *F. graminearum*. Furthermore, FgGyp1 is found to regulate the FgSnc1-mediated fusion of secretory vesicles emerging from the Golgi compartment with the cell membrane.

Previously, De Antoni et al. (2002) demonstrated *in vivo* that *GYP* genes in yeast are redundant in their functions as mutants of a single *GYP* gene remain viable. In this study, we found that a FgRab1-GAP is critical for the development of *F. graminearum* and its mutants are severely impaired in intracellular vesicle trafficking processes. There are 12 TBC domain-containing proteins in *F. graminearum* genome, and recently, the TBC domain-containing protein FgMsb3 was shown to act as a GAP for FgRab8 and is also important for *F. graminearum* pathogenicity and development (Zheng et al., 2020). When compared to the observed roles in yeast, these findings imply more elaborate biological roles of GAPs during fungal evolution. Interestingly, Δ*Fggyp1* mutant is observed to grow even slower than the Δ*Fgmsb3* mutant (Zheng et al., 2020) but the latter is more impaired in virulence than the former, suggesting a less important role of FgGyp1 in virulence than FgMsb3 in *F. graminearum*.

Gyp1 has dual functions, as a GAP for Ypt1 and as an interacting partner of Atg8 in selective autophagy (Mitter et al., 2019). For its stability and normal subcellular localization, Gyp1 (a GAP for Ypt1) needs to interact with Ypt32 (Rivera-Molina and Novick, 2009). Gyp1 localizes to the Golgi apparatus in yeast and *C. albicans* (Du and Novick, 2001; Huang et al., 2014). Moreover, Gyp1 is critically required for Golgi polarization in *C. albicans* (Huang et al., 2014). Here, we found that FgGyp1 mainly localizes to the FgKex2-positive TGN, FgBet3-positive *cis*-Golgi and FgGos1-positive medial Golgi, suggesting that FgGyp1 could be involved in various Golgi-related physiological process.

Effective vesicle trafficking of a cell depends on normal localization of Rab GTPases (Thomas et al., 2019). The targeting signal for these proteins is HVD (hypervariable domain), located at their C-termini. In this study, the C- and N-terminal regions of FgGyp1 are both required for its functions and localization in *F. graminearum*. A previous study indicated that the polarization of Gyp1 to the growth site is regulated through its phosphorylation by PKA (protein kinase A) in *C. albicans* (Huang et al., 2014); four serine residues are present at the N-terminal regulatory domain of Gyp1 and are phosphorylated by PKA. These serine residues and the Arg292 residue inside the catalytic domain of CaGyp1 are required for the polarization of CaGyp1. Here, we showed that the Arg357 residue close to the N-terminus of FgGyp1 is required for its hydrolyzing activity and functions in *F. graminearum* but not for its correct localization. We showed previously in *F. graminearum* that FgMsb3 TBC domain has an Arg681 residue that is indispensable for FgMsb3 functions, but not for the normal localization of the protein (Zheng et al., 2020). The Sec2 domain and N-terminal region of FgSec2A are dispensable for its polarized localization but required for its functions (Zheng et al., 2018a).

The role of SNAREs and Rab/Ypt GTPases in targeting vesicles to their right compartments ensures trafficking pathways specificity (Segev, 2001). In yeast, recycling of Snc1 protein ensures the continuity of the secretion pathway and Gyp1 GAP activity is needed for Snc1 recycling (Lewis et al., 2000). In yeast, Ypt6 and Ric1/Rgp1 (guanine-nucleotide exchange factor for Ypt6) are also crucial for recycling of Snc1 (Siniossoglou et al., 2000). FgRab8 and its GAP FgMsb3 are required for vesicle secretion and trafficking during exocytosis mediated by FgSnc1 (Zheng et al., 2020). We found that FgGyp1 regulates the fusion of secreted vesicles from the Golgi with the cell membrane, suggesting a conserved role of Rab/Ypt GAP in vesicle transport.

In this study, DON production is significantly increased in the Δ*Fggyp1* mutant, suggesting that FgGyp1 negatively regulates DON metabolism. The Δ*Fggyp1* mutant grows very slowly while its disease index to flowering wheat heads reaches 5.88 likely due to the increasing production of DON in this mutant. In summary, the present research identified FgGyp1 as a FgRab1 GAP that is indispensable for conidiation, virulence, growth, and DON biosynthesis in *F. graminearum*. Furthermore, FgGyp1 is critical for the regulation of FgSnc1-mediated fusion of secreted vesicles from the Golgi with the cell membrane.

## Data Availability Statement

The original contributions presented in the study are included in the article/Supplementary Material, further inquiries can be directed to the corresponding author/s.

## Author Contributions

HZ conceived and designed the experiments. HZ, QZ, ZY, YY, and DS performed the experiments. HZ wrote the manuscript. JZ, ZW, and YA critically reshaped the manuscript. All authors contributed to the article and approved the submitted version.

## Conflict of Interest

The authors declare that the research was conducted in the absence of any commercial or financial relationships that could be construed as a potential conflict of interest.

## Supplementary Material

Supplementary Figure 1 Phylogenetic relationship of Gyp1 proteins from different organisms.

Supplementary Figure 2 Generation of *FgGYP1* deletion mutants and confirmation by Southern blot analysis.

Supplementary Figure 3 FgGyp1 is dispensable for sexual reproduction.

Supplementary Figure 4 The standard curves for quantification of the DON.

Supplementary Table 1 List of the various fungal strains used in this study.

Supplementary Table 2 List of all primers involved in this study.

Supplementary Video 1 GFP-FgSnc1 protein mobility in the PH-1.

Supplementary Video 2 GFP-FgSnc1 protein mobility in the Δ*Fggyp1* mutant.
