# Corrigendum: The GTPase-Activating Protein FgGyp1 Is Important for Vegetative Growth, Conidiation, and Virulence and Negatively Regulates DON Biosynthesis in *Fusarium graminearum*

**DOI:** 10.3389/fmicb.2021.666050

**Published:** 2021-03-08

**Authors:** Qiaojia Zheng, Zhi Yu, Yanping Yuan, Danli Sun, Yakubu Saddeeq Abubakar, Jie Zhou, Zonghua Wang, Huawei Zheng

**Affiliations:** ^1^Marine and Agricultural Biotechnology Laboratory, Institute of Oceanography, Minjiang University, Fuzhou, China; ^2^College of Life Sciences, Fujian Agriculture and Forestry University, Fuzhou, China; ^3^Department of Biochemistry, Faculty of Life Sciences, Ahmadu Bello University, Zaria, Nigeria

**Keywords:** *Fusarium graminearum*, GTPase-activating protein, FgGyp1, DON, fungal development, pathogenicity

In the published article, graminearum was incorrectly spelled as graminearium in the title. The title of the original article was changed from *The GTPase-Activating Protein FgGyp1 Is Important for Vegetative Growth, Conidiation, and Virulence and Negatively Regulates DON Biosynthesis in Fusarium graminearium* to *The GTPase-Activating Protein FgGyp1 Is Important for Vegetative Growth, Conidiation, and Virulence and Negatively Regulates DON Biosynthesis in Fusarium graminearum*.

In the published article, graminearum was incorrectly spelled as graminearium in the running title. The running title was changed from *FgGyp1 in Fusarium graminearium* to *FgGyp1 in Fusarium graminearum*.

The authors apologize for these error and state that this does not change the scientific conclusions of the article in any way. The original article has been updated.

