# Supplementary material for: The GTPase-Activating Protein FgGyp1 Is Important for Vegetative Growth, Conidiation, and Virulence and Negatively Regulates DON Biosynthesis in Fusarium graminearum
Source: Front Microbiol. 2021 Jan 21;12:621519. doi: 10.3389/fmicb.2021.621519 (PMC7858271; doi:10.3389/fmicb.2021.621519)
Supplement: Supplementary Figure 1 — Phylogenetic relationship of Gyp1 proteins from different organisms. [file Data_Sheet_1.pdf]

Table S1. List of the various fungal strains used in this study.

Strain	Genotype description	Reference
PH-1	Wild type	(Cuomo et al., 2007)
Δ*Fggyp1*	FGSG_17336 deletion mutant in PH-1	(Zheng et al., 2020)
Δ*Fggyp1-C*	Δ*Fggyp1* strain expressing the FgGyp1-GFP construct	This study
FgGyp1-GFP+ FgKex2-mCherry	Δ*Fggyp1* strain expressing the FgGyp1-GFP and FgKex2-mCherry constructs	This study
FgGyp1-GFP+ FgBet3-mCherry	Δ*Fggyp1* strain expressing the FgGyp1-GFP and FgBet3-mCherry constructs	This study
FgGyp1-GFP+ mCherry-FgGos1	Δ*Fggyp1* strain expressing the FgGyp1-GFP and mCherry-FgGos1 constructs	This study
PH-1+FgTri1-GFP	PH-1 strain expressing the FgTri1-GFP construct	This study
Δ*Fggyp1*+ FgTri1-GFP	Δ*Fggyp1* strain expressing the FgTri1-GFP construct	This study
PH-1+FgTri4-GFP	PH-1 strain expressing the FgTri4-GFP construct	This study
Δ*Fggyp1*+ FgTri4-GFP	Δ*Fggyp1* strain expressing the FgTri4-GFP construct	This study
Δ*TBC*	Δ*Fggyp1* strain expressing the FgGyp1$^{\Delta TBC}$ -GFP construct	This study
Δ*N*	Δ*Fggyp1* strain expressing the FgGyp1$^{\Delta N}$-GFP construct	This study
Δ*C*	Δ*Fggyp1* strain expressing the FgGyp1$^{\Delta C}$-GFP construct	This study
R284K	Δ*Fggyp1* strain expressing the FgGyp1^{R284K}-GFP construct	This study
R357K	Δ*Fggyp1* strain expressing the FgGyp1^{R357K}-GFP construct	This study
PH-1+GFP-FgSnc1	PH-1 strain expressing the GFP-FgSnc1 construct	(Zheng et al., 2020)
Δ*Fggyp1*+ GFP-FgSnc1	Δ*Fggyp1* strain expressing the GFP-FgSnc1 construct	This study

Cuomo, C.A., Gueldener, U., Xu, J.R., Trail, F., Turgeon, B.G., Di Pietro, A., Walton, J.D., Ma, L.J., Baker, S.E., Rep, M., Adam, G., Antoniw, J., Baldwin, T., Calvo, S., Chang, Y.L., Decaprio, D., Gale, L.R., Gnerre, S., Goswami, R.S., Hammond-Kosack, K., Harris, L.J., Hilburn, K., Kennell, J.C., Kroken, S., Magnuson, J.K., Mannhaupt, G., Mauceli, E., Mewes, H.W., Mitterbauer, R., Muehlbauer, G., Munsterkotter, M., Nelson, D., O'donnell, K., Ouellet, T., Qi,

W.H., Quesneville, H., Roncero, M.I.G., Seong, K.Y., Tetko, I.V., Urban, M., Waalwijk, C., Ward, T.J., Yao, J.Q., Birren, B.W., and Kistler, H.C. (2007). The Fusarium graminearum genome reveals a link between localized polymorphism and pathogen specialization. *Science* 317**,** 1400-1402.

Zheng, H., Li, L., Yu, Z., Yuan, Y., Zheng, Q., Xie, Q., Li, G., Abubakar, Y.S., Zhou, J., Wang, Z., and Zheng, W. (2020). FgSpa2 recruits FgMsb3, a Rab8 GAP, to the polarisome to regulate polarized trafficking, growth and pathogenicity in Fusarium graminearum. *New Phytol* 10.1111/nph.16935.

Table S2. List of all primers involved in this study.

Primer	Sequence (5′→3′)	Application
FGSG_17336AF	GCGAGTTAGCGGACAACC	*FgGYP1* deletion
FGSG_17336AR	TTGACCTCCACTAGCTCCAGCCAAGCCTCATCATCCTCATAGCCCATC	
FGSG_17336BF	GAATAGAGTAGATGCCGACCGCGGGTTTGTTCCTCCAGAGCCTTCC	
FGSG_17336BR	CCCGTTTACTACACCACCC	
FGSG_17336OF	GCTCGCTCCTCGCATAAT	
FGSG_17336OR	ACCACTTGCTGGGTGTCG	
FGSG_17336UA	TTTTCAGCGTCCTATGTAGCC	
H853	GACAGACGTCGCGGTGAGTT	
Fg17336CF	agggaacaaaagctgggtaccTGCCTCTGATGACAGAACCT	FgGyp1-GFP
Fg17336GR	GCCGCCGCCGCCGCCAAGCTTTAGTTGTAAATTCGTGACGG	
FgGyp1-R284K-R1	AGCAGCAGTTGCCAGGTAATGGCCTTAACCTCTTGCGGCACACCAG	For R284K mutation
FgGyp1-R284K-F1	TGGTCTGGTGTGCCGCAAGAGGTTAAGGCCATTACCTGGCAACTGCTG	
FgGyp1-R357K-R1	TACAGCTCAATATGCGGGTTGGTCTTGGGTACATCGATGCTGATTT	For R357K mutation
FgGyp1-R357K-F1	ACCAAATCAGCATCGATGTACCCAAGACCAACCCGCATATTGAGCT	
17336-TBC-R1	CGGCACACCAGACCAGGCGA	For TBC domain deletion
17336-TBC-F1	GGCAACTCGCCTGGTCTGGTGTGCCGGACATGGATTTCCAGGAGAT	
17336ZR	CATGGTTCGATGACGAGAAGTGA	For C/N-terminal deletion of FgGyp1
17336-N-F	TCACTTCTCGTCATCGAACCATGCAAGAGGTTCGCGCCATTAC	
17336queC-R	GCCGCCGCCGCCGCCAAGCTTGAGGAGCTTATCGGACCATTT	
FgBet3CF	agggaacaaaagctgggtaccTCCCTTTCTCAGCCGTAG	FgBet3-mCherry
FgBet3CR	gcccttgctcaccataagcttGTCATCCTCAGGTGGCAGTT	
FgGyp1-MBP-F	ATCGAGGGAAGGATTTCAGAATTCATGTGGTCATCTTCTGGAAG	For MBP-FgGyp1
FgGyp1-MBP-R	GCCAGTGCCAAGCTTGCCTGCAGTCATAGTTGTAAATTCGTGA	
FgGyp1-TBC-MBP-F	ATCGAGGGAAGGATTTCAGAATTCATGCAAGAGGTTCGCGCCATTAC	For MBP-TBC

FgGyp1-TBC-M	GCCAGTGCCAAGCTTGCCTGCAGTCAGA
BP-R	GGAGCTTATCGGACC

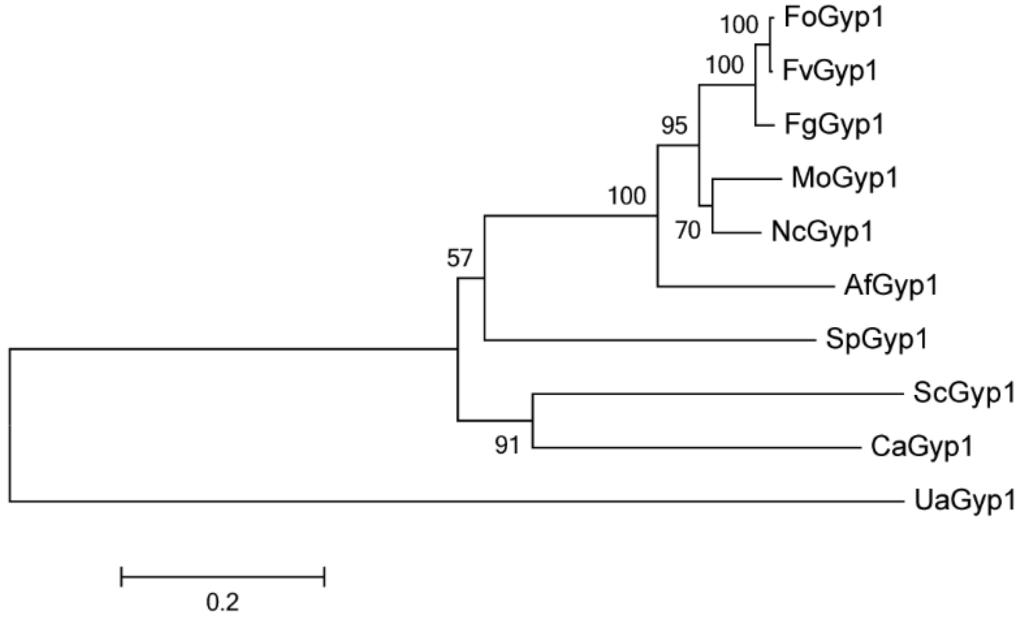

Figure S1. Phylogenetic relationship of Gyp1 proteins from different organisms
Sequence alignments were performed using the Clustal X 1.83 program and the calculated phylogenetic tree was viewed using Mega 6 program. A neighbour-joining tree with 10000 bootstrap replicates between the Gyp1 homologs in the different organisms.

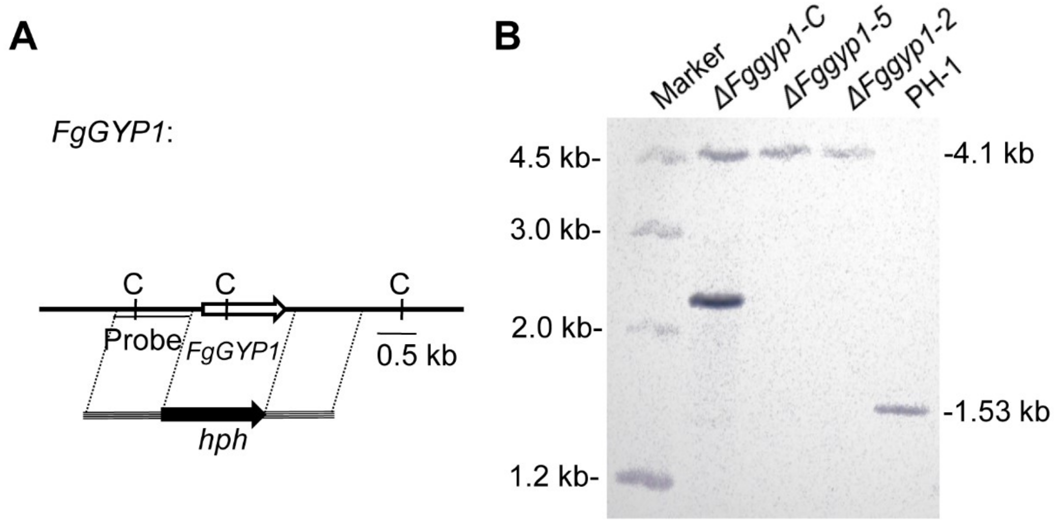

Figure S2. Generation of *FgGYP1* deletion mutants and confirmation by Southern blot analysis

(A) The split-marker approach was used to delete the *FgGYP1* gene. Genomic DNAs were extracted from PH-1 and the putative transformants. Schematic diagram of the genomic regions of *FgGYP1* and *hph* genes

(B) Targeted gene deletion of *FgGYP1, Cla*I (C) digested DNAs showed a 1.53 kb band in PH-1 and a 4.10 kb band in the mutants.

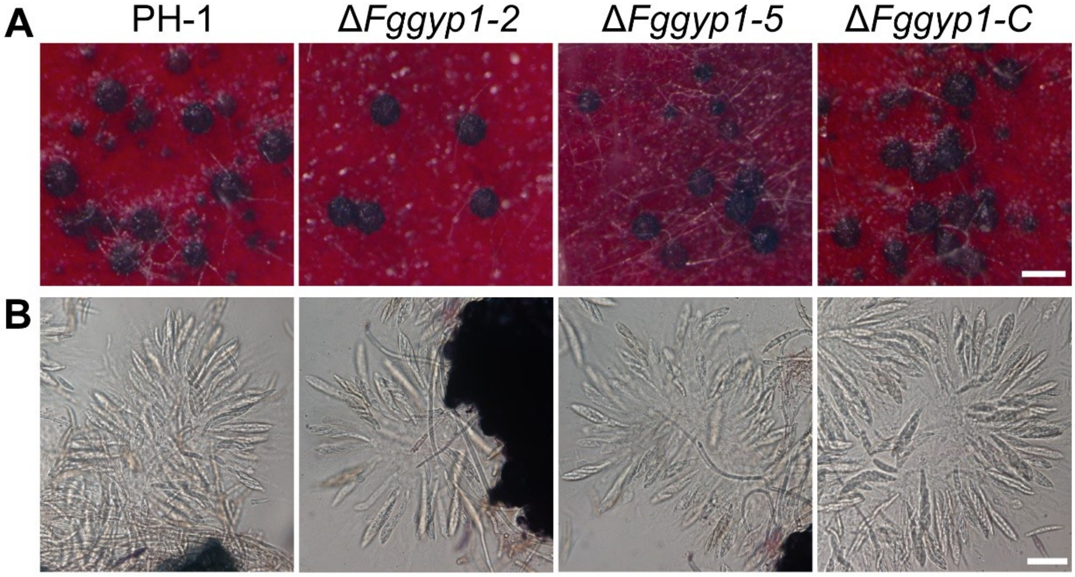

Figure S3. FgGyp1 is dispensable for sexual reproduction

(A) FgGyp1 is not required for perithecium formation. Bar = 200 μm.

(B) FgGyp1 is not required for ascospore formation. Bar = 50 μm.

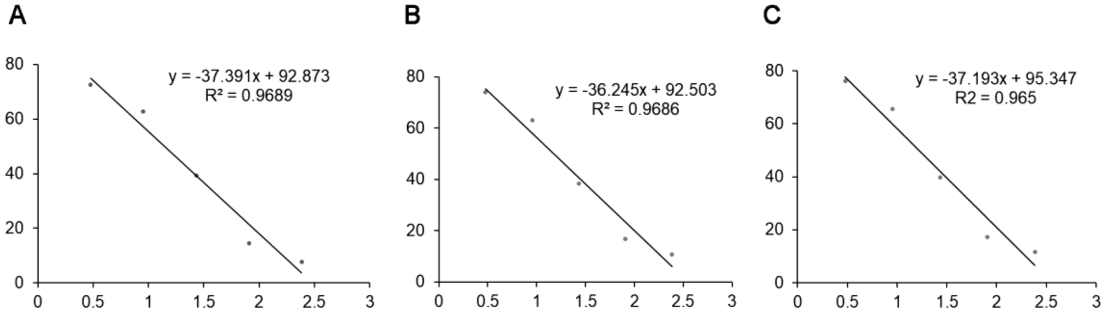

Figure S4. The standard curves for quantification of the DON
(A-C) The standard curves of three independent experiments for quantification of the DON. x: logarithm of the standard solution concentration. y: percentage of the standard solution absorbance.
